# Angiotensin II Promotes SARS-CoV-2 Infection via Upregulation of ACE2 in Human Bronchial Cells

**DOI:** 10.3390/ijms23095125

**Published:** 2022-05-04

**Authors:** Ilaria Caputo, Brasilina Caroccia, Ilaria Frasson, Elena Poggio, Stefania Zamberlan, Margherita Morpurgo, Teresa M. Seccia, Tito Calì, Marisa Brini, Sara N. Richter, Gian Paolo Rossi

**Affiliations:** 1 Specialized Center for Blood Pressure Disorders-Regione Veneto and Internal Emergency Medicine Unit, Department of Medicine-DIMED, University of Padua, 35128 Padua, Italy; ilaria.caputo@unipd.it (I.C.); brasilina.caroccia@unipd.it (B.C.); stefania.zamberlan@studenti.unipd.it (S.Z.); teresamaria.seccia@unipd.it (T.M.S.); 2 Department of Molecular Medicine-DMM, University of Padua, 35121 Padua, Italy; ilaria.frasson@unipd.it (I.F.); sara.richter@unipd.it (S.N.R.); 3 Department of Biology, University of Padua, 35131 Padua, Italy; elena.poggio@unipd.it (E.P.); marisa.brini@unipd.it (M.B.); 4 Department of Pharmaceutical and Pharmacological Sciences, University of Padua, 35131 Padua, Italy; margherita.morpurgo@unipd.it; 5 Department of Biomedical Sciences, University of Padua, 35131 Padua, Italy; tito.cali@unipd.it

**Keywords:** angiotensin-converting enzyme 2, angiotensin II, severe acute respiratory syndrome coronavirus 2, angiotensin-converting enzyme inhibitor, angiotensin receptor blocker, COVID-19

## Abstract

Blockers of the renin-angiotensin system (RAS) have been reported to increase the angiotensin converting enzyme (ACE)2, the cellular receptor of SARS-CoV-2, and thus the risk and course of COVID-19. Therefore, we investigated if angiotensin (Ang) II and RAS blockers affected ACE2 expression and SARS-CoV-2 infectivity in human epithelial bronchial Calu-3 cells. By infectivity and spike-mediated cell–cell fusion assays, we showed that Ang II acting on the angiotensin type 1 receptor markedly increased *ACE2* at mRNA and protein levels, resulting in enhanced SARS-CoV-2 cell entry. These effects were abolished by irbesartan and not affected by the blockade of ACE-1-mediated Ang II formation with ramipril, and of ACE2- mediated Ang II conversion into Ang 1-7 with MLN-4760. Thus, enhanced Ang II production in patients with an activated RAS might expose to a greater spread of COVID-19 infection in lung cells. The protective action of Angiotensin type 1 receptor antagonists (ARBs) documented in these studies provides a mechanistic explanation for the lack of worse outcomes in high-risk COVID-19 patients on RAS blockers.

## 1. Introduction

The angiotensin-converting enzyme-2 (ACE2) is the cellular receptor of the severe acute respiratory syndrome corona virus 2 (SARS-CoV-2) causing COVID-19 [1]. Studies reporting increased ACE2 expression with angiotensin (Ang)-converting enzyme-1 (ACE-1) inhibitors (ACEIs) and Ang type 1 receptor (AT1R) antagonists (ARBs) [2,3,4,5] suggested that renin-angiotensin system (RAS) blockers could worsen the prognosis of COVID-19 patients [6,7]. Current evidence on increased ACE2 expression with RAS blockers is derived from studies of renal tubular cells [8] and limited experimental studies [2,3]. Nonetheless, this contention raised enormous concern in the medical community because millions of patients who have an activated RAS are currently receiving these life-saving drugs worldwide [9,10].

Accumulating data, thereafter, suggested that RAS blockers could be neutral [11] or even beneficial in COVID-19 patients [12] despite their worse prognosis because of arterial hypertension, heart failure, kidney diseases, and/or a prior myocardial infarction. It was also contended that these drugs could protect the lung from acute respiratory failure, as they do against severe pneumonia in fragile patients [13,14,15]. Moreover, RAS blockers were shown to be beneficial both experimentally [16] and clinically [9,14,15] when the RAS is activated, not only because they blunt the AT1R-mediated vasoconstrictive pro-inflammatory actions, but also because they enhanced its protective arm comprising ACE2, Ang 1-7, and the Mas receptor [17,18]. Accordingly, clinical trials that randomized COVID-19 patients to continue or suspend treatment with RAS blockers reported similar rates of acute hospitalization [19] and equal mean number of days alive and out of the hospital [20]. 

The knowledge of the effects of Ang II on ACE2 expression and activity in human lung cells, one of the main targets of SARS-CoV-2, is, therefore, a fundamental piece of information to obtain. We, therefore, set up this study to answer several simple yet unanswered questions: (*i*) Does Ang II increase ACE2 expression in human endothelial and airway epithelial Calu-3 cells? (*ii*) Can ACEIs or ARBs blunt or enhance the effect of Ang II on ACE2 expression? (*iii*) Does Ang II enhance the airway epithelial cell SARS-CoV-2 infection and spread? (*iv*) Do these effects occur via the spike protein interaction with ACE2 and downstream activation of the TMPRSS2 protease? 

To answer these questions, we measured the mRNA absolute copy number of *AGTR1* and *ACE2* with a state-of-the-art digital droplet quantitative PCR in Calu-3 cells. This is a stable ACE2 expressing cell line derived from human bronchial epithelium, which was widely used to mechanistically investigate SARS-CoV-2 cell entry and replication. We also investigated the functional relevance of ACE2 changes by measuring the effect of Ang II in the presence/absence of ACEIs or ARBs on virus entry and infection in Calu-3 cells infected with wild-type SARS-CoV-2 or a spike protein-expressing pseudotyped virus (pseudovirus). Furthermore, we used a dual split protein-based spike-mediated cell–cell fusion assay mimicking the viral attachment and the entry steps to determine the effect of Ang II on spike protein-mediated infection and spread.

## 2. Results

### 2.1. ACE2 and AGTR1 Expression in HUVEC and Calu-3 Cells 

We found that *ACE2* and *AGTR1* mRNA copies were abundantly expressed in Calu-3 cells and were barely detectable in HUVEC (Figure S1, panels a and b). Therefore, we performed all further experiments with Ang II and its antagonists in Calu-3 cells.

### 2.2. Ang II Upregulates ACE2 Gene Expression via AT1R Signalling

**Figure 1:**
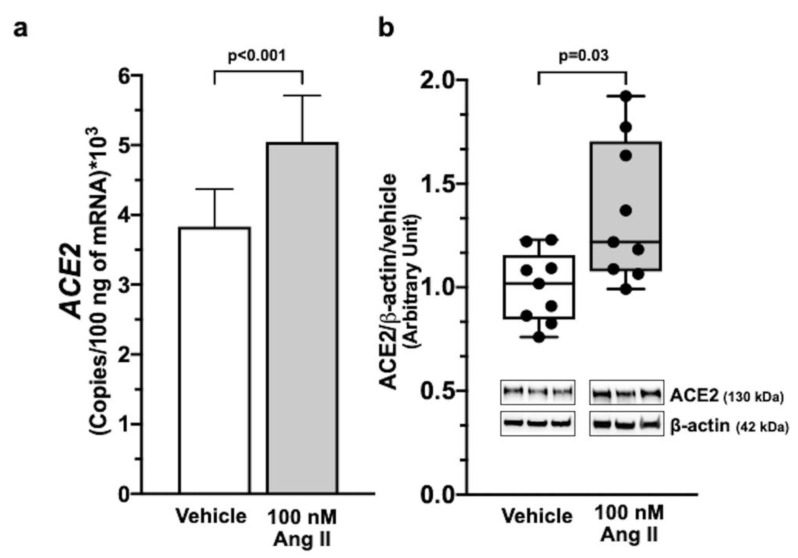
Ang II-induced ACE2 expression in Calu-3 cells. Ang II increased *ACE2* mRNA and protein levels in Calu-3 cells as analyzed by digital droplet PCR (ddPCR) and Western blotting analysis. Panel (**a**) shows *ACE2* gene expression after a 12 h exposure of Calu-3 cells to 100 nM Ang II (n = 5 in duplicate; median ± SEM). Immunoblots and histograms show that 100 nM Ang II increased ACE2 protein levels in Calu-3 cells after 48 h exposure. Data were normalized on vehicle and β-actin as a loading control (panel (**b**)) (n = 3 in triplicate; median ± QR).

**Figure 2:**
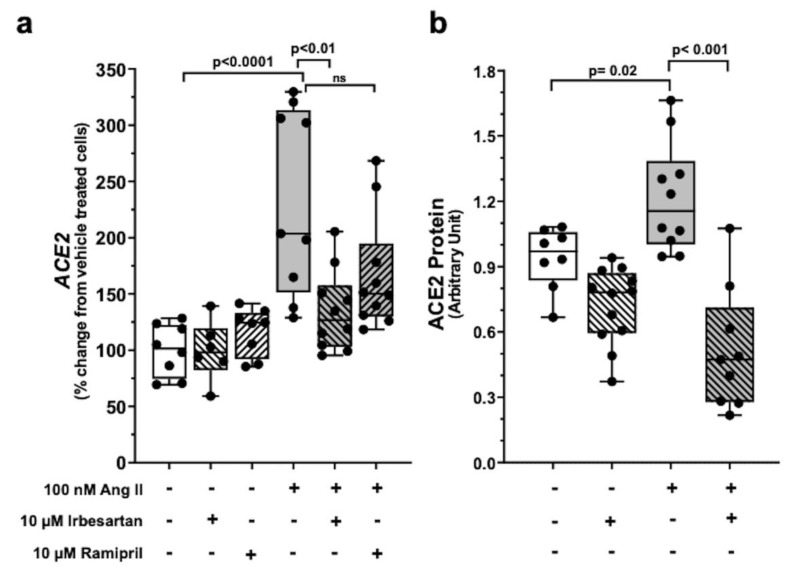
Ang II upregulation of ACE2 expression in Calu-3 cells occurs via AT1R signaling. Ramipril, *per se*, had no effect on *ACE2* mRNA abundance and neither abolished the stimulatory effect of Ang II on *ACE2* mRNA (panel (**a**)) in Calu-3 cells. Irbesartan, *per se*, had no effect on *ACE2* mRNA but abolished the stimulatory effect of Ang II on *ACE2* mRNA (panel (**a**)) in Calu-3 cells. Data were measured as copies/100 ng total mRNA and then normalized vs. vehicle-treated cells (at least n = 4 in duplicate; median ± QR). Histograms from densitometric analysis of immunoblotting show that 100 nM Ang II increased ACE2 protein levels in Calu-3 cells after 48 h exposure, and again irbesartan abolished this effect. Data were normalized on vehicle and β-actin as a loading control (panel (**b**)) (at least n = 3 in triplicate; median ± QR).

Ang II at 100 nM increased *ACE2* mRNA (*p* < 0.001) and protein (*p* < 0.05) in Calu-3 cells over vehicle-treated cells (Figure 1, panel a and panel b). The ACE-1 inhibitor ramipril and the ARB irbesartan had no effect on basal *ACE2* mRNA and protein levels at a 100-fold higher concentration (10 µM). However, irbesartan abolished the effect of Ang II (Figure 2, panels a and b), while ramipril was ineffective. 

### 2.3. Exclusion of Ang 1-7-Mediated Effect on ACE2 in Calu-3 cells

ACE2 converts *in vitro* Ang II into Ang 1-7, which counteracts Ang II effects in several models. Therefore, we wondered if Ang 1-7 could alter the action of Ang II in Calu-3 cells. Results showed that increasing concentrations (from 10 nM to 10 µM) of Ang 1-7 did not affect *ACE2* expression (Figure S2, panel a). Since Ang II might also enhance its own conversion into Ang 1-7 by markedly increasing ACE2, we repeated the experiments with ACE2 inhibitor MLN-4760. The latter *per se* had no effect on *ACE2* mRNA; furthermore, it by no means affected the Ang II-induced increase in *ACE2* (Figure S2, panel b). 

### 2.4. Ang II and Irbesartan Modulate SARS-CoV-2 Infection in Human Lung Cells

**Figure 3:**
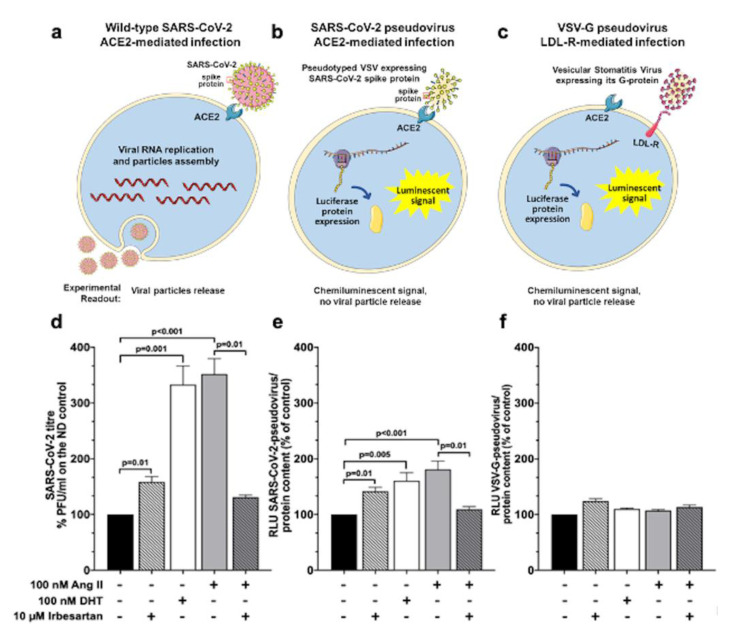
Effects of Ang II and irbesartan on SARS-CoV-2 pseudovirus and virus infection in human lung cells. The scheme depicts 3 different assays that were used to evaluate the effect of Ang II and irbesartan on SARS-CoV-2 infection in Calu-3 cells: with wild-type SARS-CoV-2 virus (panel (**a**)), with pseudotyped vesicular stomatitis virus (VSV) expressing SARS-CoV-2 spike protein (panel (**b**)) and with a genetically modified VSV that binds to the low-density lipoprotein receptor and not to ACE2 (panel (**c**)). Please note that the size of Calu-3 cells and viruses is shown for the purpose of graphical illustration and does not correspond to reality. Calu-3 cells were pre-treated for 48 h with 100 nM dihydrotestosterone (DHT), 100 nM Ang II, 10 µM irbesartan alone, or on top of Ang II prior to infection with the SARS-CoV-2 virus (panel (**d**)), with the SARS-CoV-2-pseudovirus (panel (**e**)) or with the VSV-G-pseudovirus (panel (**f**)). SARS-CoV-2 infection in Calu-3 cells indicated that DHT and Ang II treatment enhanced viral infection (333% and 352%, respectively). Irbesartan slightly (by 158%) promoted viral infection over controls but it drastically blunted the viral infection induced by Ang II (panel (**d**), n = 3 in triplicate; mean ± SEM). SARS-CoV-2-pseudovirus infection in Calu-3 cells showed that both DHT stimulation on TMPRSS2 protease and Ang II effect on ACE2 enhanced pseudoviral entry (160% and 180%, respectively). A similar effect was obtained in presence of irbesartan (142%). The concomitant administration of Ang II and irbesartan diminished the percentage of infection (109%) with respect to the administration of Ang II alone (panel (**e**), n = 3 in triplicate; mean ± SEM). VSV-G-pseudovirus infection in Calu-3 cells showed negligible effects upon DHT and Ang II treatment (109% and 107%, respectively), whereas irbesartan positively influenced pseudoviral infection (124%). The concomitant administration of Ang II and irbesartan showed a reduction in pseudoviral infection (114%) with respect to the administration of Ang II or irbesartan (panel (**f**), n = 3 in triplicate; mean ± SEM).

We measured the effects of Ang II on the amount of new infective particles (viral titre) 48 h post-infection in a plaque reduction assay (Figure 3, panels a and d) to determine if the Ang II-induced ACE2 increase affected the SARS-CoV-2 wild-type virus entry in Calu-3 cells. Thus, cells treated with Ang II alone, or on top of irbesartan, were exposed to SARS-CoV-2 at MOI 0.05. We also tested the effect of the peptide *vis-a-vis* that of the known TMPRSS2 protease inducer DHT [21] to determine if Ang II could augment the cell receptors/mechanisms exploited by SARS-CoV-2 to enter the cells. Results showed that both Ang II and DHT enhanced over three-fold (352% and 333%, respectively) SARS-CoV-2 viral titer, indicating that both the Ang II-induced expression of ACE2 and the induction of TMPRSS2 similarly increased SARS-CoV-2 cell entry (Figure 3, panel d). Importantly, irbesartan drastically blunted the Ang II-induced augmented viral infection (Figure 3, panel d), indicating that Ang II-induced increased SARS-CoV-2 cell infection involves AT1R. Of note, irbesartan *per se* also slightly (by 158%) promoted viral infection over controls.

To investigate if what was observed with the SARS-CoV-2 wild-type virus depended on virus entry in Calu-3 cells, we also used the SARS-CoV-2 pseudovirus, a luciferase-expressing vesicular stomatitis virus (VSV) pseudotyped with SARS-CoV-2 spike protein (Figure 3, panel b). This pseudovirus recapitulates the entry step of SARS-CoV-2 in cells (Figure 3, panel b). After challenging Calu-3 cells with SARS-CoV-2 pseudovirus at MOI 0.05, we observed that both Ang II and DHT enhanced by 160% and 180%, respectively, pseudoviral entry, as shown by an increased luciferase signal over controls (Figure 3, panel e). Importantly, irbesartan counteracted the Ang II- promoted pseudovirus cell entry (Figure 3, panel e), although it also enhanced pseudoviral entry by itself, albeit much less effectively than Ang II and DHT.

### 2.5. SARS-CoV-2 Entry in Human Lung Cells Involves ACE2, TMPRSS2, and Spike Protein

We further examined the mechanisms of virus infection using the luciferase expressing vesicular viral stomatitis (VSV) with its own natural attachment glycoprotein G protein [22] to characterize the cell entry-enhancing mechanisms of Ang II, DHT, and irbesartan. Instead of ACE2, VSV recognizes the low-density lipoprotein receptor (LDL-R) [23] and, therefore, allows the assessment of ACE2- and spike protein-independent cell entry (Figure 3, panel c).

Of interest, both DHT and Ang II had negligible effects on VSV-derived luciferase signal (Figure 3, panel f), indicating that these compounds enhance the wild-type SARS-CoV-2 virus and SARS-CoV-2 pseudovirus infection via attachment and processing the spike protein relative to ACE2 and ensuing TMPRSS2 activation. 

Irbesartan slightly (by 124%) stimulated VSV-G-pseudovirus infection, but co-exposure to Ang II did not affect its action (Figure 3, panel f). Along with the fact that irbesartan did not alter *ACE2* mRNA and protein levels in Calu-3 cells (Figure 2, panels a and b), these data may suggest that the slight increase in bronchial epithelial cell permissiveness to viruses seen with the ARB does not involve ACE2 and the spike protein.

**Figure 4:**
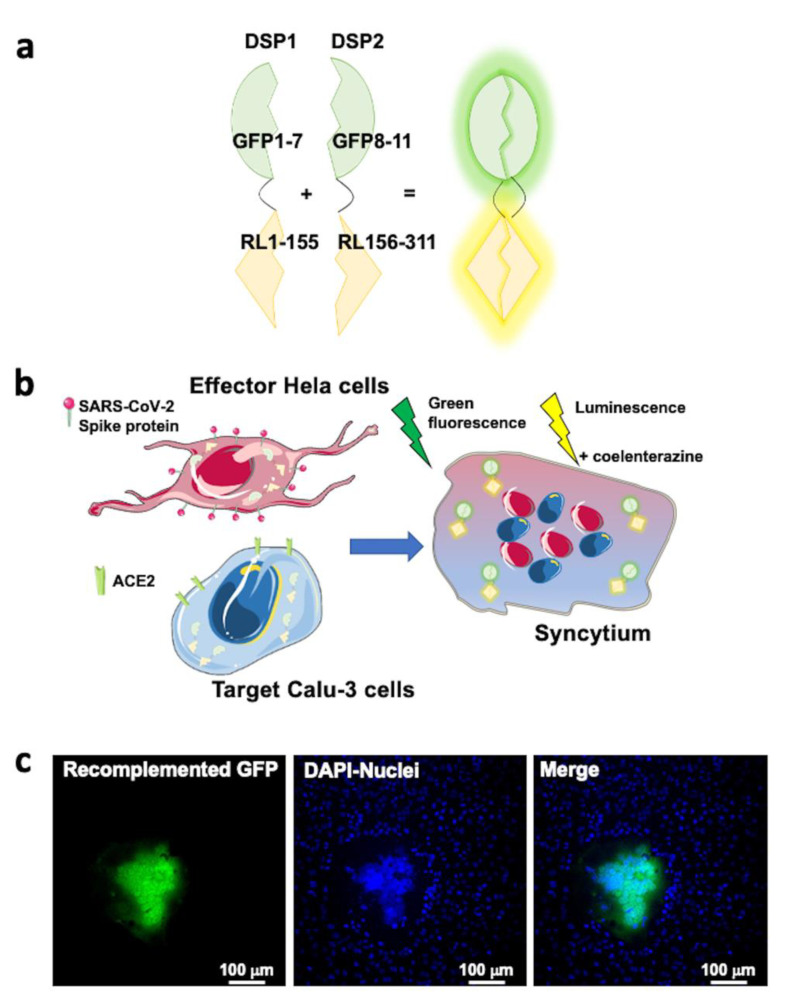
SARS-CoV-2 S protein, ACE2, and TMPRSS2 are necessary for viral entry as reported by cell–cell fusion assay. Panel (**a**): The Dual Split Protein (DSP) reporters: DSP1 consisting in the first seven ß-strands of the green fluorescent protein (GFP) fused together with the first 155 amino acid residues of the Renilla luciferase enzyme (RL), DSP2 consisting in the remaining part of both GFP and RL. The DSPs alone do not show any luminescent or fluorescence emission. Panel (**b**): Hela cells expressing the SARS-CoV-2 spike protein after transfection were co-cultured with target cells, Calu-3 cells. Calu-3 cells are expressing endogenously the angiotensin-converting enzyme 2 (ACE2) and the cell surface transmembrane serine protease 2 (TMPRSS2). During the co-culture, the spike protein exogenously expressed by Hela cells interacts with ACE2 and is primed by TMPRSS2 on Calu-3 cells, initiating the fusion process. The fusion event between the effector and the target cell membranes results in the formation of a syncytium when mixing two cytoplasmic contents. Only within syncytia, DSP1 and DSP2 can self-associate restoring a fully fluorescent GFP and a fully functional RL. Panel (**c**): Representative images of the co-culture between Hela cells expressing SARS-CoV-2 spike protein and DSP2 and Calu-3 cells expressing DSP1. Images were acquired with ZEISS LSM700 confocal microscope with an EC “Plan-Neofluar” 20x/0.50 M27 objective upon illumination with laser at the wavelength of 488 nm to detect the recomplemented GFP signal and 405 nm to detect the cell nuclei stained with Hoechst 33342 (ThermoFischer, cat. #H3570). In panel (**c**), from the merge of the two fluorescent signals, it is possible to appreciate the formation of a syncytium with the recomplemented GFP diffused in the cytosol and the aggregation of several cell nuclei.

**Figure 5:**
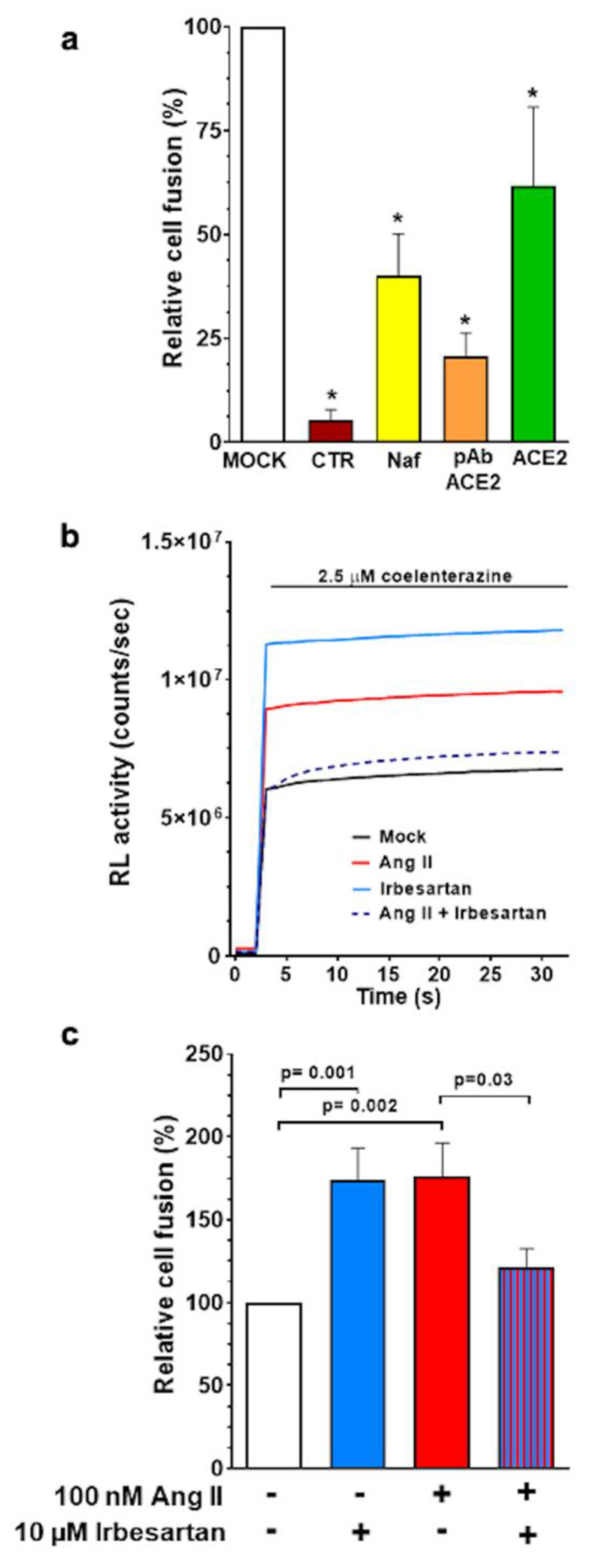
Antihypertensive drugs modulate SARS-CoV-2 cell entry mediated by the spike protein as assessed with the cell–cell fusion assay. Panel (**a**): The cell fusion observed with the cell–cell fusion assay is directly dependent on (1) the presence of the SARS-CoV-2 spike protein, since no significant luminescent signal is detected when Hela cells expressing only DSP2 and Calu-3 cells expressing DSP1 were co-cultured (CTR, negative control conditions); (2) the presence of ACE2, since the fusion efficiency was drastically reduced when Calu-3 cells were incubated with 2µg/mL of a polyclonal antibody targeting ACE2 (pAb-ACE2), or with soluble recombinant ACE2 protein for at least 2 h before the co-culture with Hela cells; (3) the presence of active TMPRSS2, since the fusion efficiency was drastically reduced when Calu-3 cells were incubated with 10 nM Nafamostat mesylate (Naf) at least 2 h before the addition of Hela cells. Histograms are the mean ± SEM of the relative cell fusion from at least 3 independent experiments (* *p* < 0.05 *vs.* mock). Panel (**b**): Representative traces of RL activity detected with a luminometer in cell–cell fusion assay. Coelenterazine, the membrane permeable substrate for RL, was added to the cells and the fusion efficiency was directly measured as a luminescent signal (counts/seconds). For each well, after 3 s, 2.5 μM coelenterazine was added and the luminescent signal was recorded for 30 s. The relative cell–cell fusion values were calculated by normalizing the average RL activity of each condition to the average RL activity of the co-culture in mock condition, which was set to 100%. Panel (**c**): Target cells, Calu-3 cells, were plated on 96 wells plate and incubated with 10 μM irbesartan, 100 nM Ang II either alone or in co-incubation, as indicated. In mock conditions, cells were incubated with the drug vehicle for 48 h before co-culture with effector Hela cells. Histograms are the mean ± SEM of the relative RL activity from at least 4 independent experiments.

The specificity of spike dependent cell entry-enhancing action of Ang II and irbesartan was further investigated in a cell–cell fusion assay where the presence of viral particles is simulated by effector cells expressing the SARS-CoV-2 spike protein on their plasma membrane surface. Hela cells transfected to express the spike protein were, therefore, co-cultured with target Calu-3 cells (Figure 4) to explore the endosomal-independent viral entry and to quantify the process of spike protein/ACE2 mediated virus entry. Calu-3 cell incubation with three putative inhibitors of this process -nafamostat mesylate, antibodies against ACE2 (pAB anti ACE2), and soluble ACE2 recombinant protein- blunted SARS-CoV-2 spike-mediated cell–cell fusion compared to mock conditions (Figure 5). These results indicated the obligatory role of ACE2 and TMPRSS2 for viral infection and cell fusion. They suggest that ACE2 accessibility on the cell membrane surface is key for cell fusion efficiency, as pAB anti-ACE2s were particularly effective. 

In these experiments, the exposure of the co-cultured cells to either 100 nM Ang II or 10 μM irbesartan increased cell fusion efficiency compared to mock conditions (Figure 5, panels b and c), thus confirming what was observed in the infection assay with pseudovirus and wild-type SARS-CoV-2 (Figure 3, panels d and e). Importantly, irbesartan abolished the enhancement of cell–cell fusion efficiency induced by Ang II, indicating that AT1R blockade reduces spike protein interaction with ACE2.

## 3. Discussion

Seven decades after the isolation and chemical synthesis of Ang II and the identification of its multiple physiological effects [24], we herein discovered a novel action of the peptide. This action is key for understanding SARS-CoV-2 viral infection in patients suffering from conditions featuring an activated RAS, as severe arterial hypertension, renovascular hypertension, liver cirrhosis with ascites, left ventricular hypertrophy [25], heart failure, kidney diseases, and myocardial infarction. In these conditions, RAS blockers, such as ACEIs or ARBs, are life-saving drugs [9,11,12,14,15], but they were suggested to be detrimental in COVID-19 patients because they increase ACE2 expression [6,7]. Whether this contention has a mechanistic basis or simply reflects an artefactual association created by the fact that RAS blockers are prescribed to patients already at higher risk of death remained altogether unclear.

Unambiguous evidence points to the fact that the lung is the entry site and a major target of SARS-CoV-2 infection. Accordingly, human bronchial epithelial Calu-3 cells have been used to explore the mechanisms of SARS-CoV-2 infection [26,27]. Importantly, lung cells are exposed to high concentrations of Ang II because the lung vascular endothelium is a major site of the conversion of Ang I to Ang II by ACE-1. 

We found that Calu-3 cells express abundant *ACE2* and *AGTR1* mRNA amounts, which is at variance with umbilical vein endothelial cells (HUVEC) where these genes were barely detectable (Supplemental Figure S1, panels a and b). Thus, we identified Calu-3 cells as suitable to investigate the role of RAS in SARS-CoV-2 infection, at variance with human endothelial cells, even though the latter can also be a target of SARS-CoV-2 [28]. 

We then showed that Ang II, at concentrations mimicking the pathophysiological levels of the peptide in COVID-19 patients [29], potently enhanced ACE2 expression (Figure 1) by acting via AT1R (Figure 2). These results can explain why patients with diseases featuring an activated RAS, who are at higher risk of death, showed no worse COVID-19 outcomes, and even a better prognosis when on an RAS blocker, as documented in observational studies, in randomized trials, and in meta-analyses [19,20,30,31,32,33]. 

Of note, in our studies, ACEI ramipril did not blunt Ang II-induced upregulation of *ACE2* mRNA in Calu-3 cells (Figure 2, panel a), suggesting that an autocrine production of Ang II does not contribute to the potent stimulatory effect of exogenous Ang II under our experimental conditions. Yet, from a clinical standpoint, this *in vitro* finding by no means speaks against a beneficial effect of ACEIs *in vivo*, where they display well-documented blunting effects on Ang II formation. 

Experiments with the wild-type SARS-CoV-2 virus, a human vesicular stomatitis virus (VSV), and a genetically modified VSV (Figure 3, panels a–f) collectively showed the functional consequences of Ang II-induced ACE2 increase: In the presence of Ang II or the TMPRSS2 protease inducer DHT [21], Calu-3 cells showed a prominent increase in new infective wild-type SARS-CoV-2 particles (viral titer) (Figure 3, panel d), indicating that these agents promote SARS-CoV-2 cell entry, albeit with different mechanisms. The luciferase-expressing vesicular stomatitis virus (VSV) pseudotyped with SARS-CoV-2 spike protein (SARS-CoV-2 pseudovirus) mimics the early steps of SARS-CoV-2 entry into Calu-3 cells [34,35] and, furthermore, can be detected by measuring the cell luciferase signal (Figure 3, panel e). As observed with wild-type SARS-CoV-2 virus, both Ang II and DHT enhanced pseudoviral cell entry with mechanisms that were counteracted by irbesartan (Figure 3, panel e). Considering that ARBs are prescribed in the setting of an activated RAS, these actions of irbesartan are quite important (Figure 3, panel d).

Moreover, with a luciferase expressing VSV pseudovirus [22] that recognizes the low-density lipoprotein receptor (LDL-R), we explored the ACE2-independent cell entry-enhancing action of different molecules [23]. Both DHT and Ang II had negligible effects on the VSV-derived luciferase signal in this setting (Figure 3, panel f), indicating that the two compounds enhance wild-type SARS-CoV-2 virus and SARS-CoV-2 pseudovirus infection via the spike protein attachment to ACE2 and downstream activation of TMPRSS2. 

Irbesartan slightly stimulated VSV-G-pseudovirus infection but did not alter *ACE2* mRNA in Calu-3 cells; moreover, co-exposure to Ang II, which enhanced *ACE2* expression (Figure 1, panel a), did not affect pseudovirus infection (Figure 3, panel f). Thus, these results suggest that ARBs, as irbesartan, can enhance cellular permissiveness to some viruses by acting independently of ACE2. Notably, the concomitant presence of irbesartan and Ang II, mimicking what occurs *in vivo* in patients treated with ARBs inhibitor, did not augment SARS-CoV-2 (or pseudovirus) infection in human lung cells.

The use of a cell fusion assay to explore the effect of Ang II on the SARS-CoV-2 spike protein-mediated cell entry confirmed the results obtained by the viral infection assay and is supported by multiple observations: (*i*) the lack of detectable luminescence signal in control conditions, i.e., when Calu-3 cells expressing DSP1 were co-incubated with Hela cells expressing only DSP2; (*ii*) the markedly blunted fusion efficiency with exposure to different inhibitors of ACE2- and spike protein-mediated cell entry, including the potent inhibitor of TMPRSS2 nafamostat mesylate [36,37,38], a polyclonal antibody targeting ACE2, and a soluble ACE2 recombinant protein (Figure 5, panel a). 

Taken together, these experiments demonstrate the obligatory role of the SARS-CoV-2 spike protein for cell–cell fusion efficiency and provide evidence that Ang II markedly enhanced Calu-3 cell infection by acting via AT1R, as its effects of cell fusion efficiency were blunted by irbesartan (Figure 5, panels b and c).

Hence, enhanced Ang II levels can raise the expression of ACE2 in tissues as the lung bronchial epithelial cells, thus increasing binding of the SARS-CoV-2 virus and facilitating both the infection and its spread. AT1R blockade, by counteracting cell fusion efficiency induced by Ang II, could protect from SARS-CoV-2 infection (Figure 5, panel c) in patients with cardiovascular comorbidities and RAS activation either because of the disease itself and/or because of concurrent treatment with drugs that stimulated it as, for example diuretics, vasodilators and sympathomimetic agents. 

Under these conditions, both ACEI and ARBs can, therefore, prevent the Ang II-induced enhanced expression of ACE2. Accordingly, while the activation of the RAS can be a detrimental mechanism contributing to excess SARS-CoV-2 morbidity and mortality, blunting this system can ameliorate the course of COVID-19. 

Finally, the present study provided novel information on the so-called “protective” branch of RAS in COVID-19: our results showed that Ang 1-7 did not affect *ACE2 per se* (Supplemental Figure S2, panel a) under baseline conditions or when *ACE2* was upregulated by Ang II. In fact, the upregulation of *ACE2* induced by Ang II was not affected by blocking its activity with MLN-4760 (Supplemental Figure S2, panel b) and, thus, involved a direct action on AT1R with no enhanced conversion of Ang II to Ang 1-7.

In conclusion, by acting via AT1R, Ang II potently upregulates the expression of ACE2 and subsequently SARS-CoV-2 infection by enhancing virus entry and the spread of the infection in human bronchial epithelial cells. This is a fundamental novel piece of information that supports the hypothesis that an activated RAS increases the susceptibility to SARS-CoV-2 infection and aggravates the prognosis of COVID-19 patients. The fact that the blockade of the AT1R with ARBs abolishes all these untoward actions of Ang II can explain the protective effects of these agents in patients at high risk for several cardiovascular and renal disease conditions who are being treated with these drugs because of an activated RAS. 

## 4. Materials and Methods

### 4.1. Cell Cultures 

Since SARS-CoV-2 infection causes pneumonia and endothelial damage, we used Calu-3 (HTB55; ATCC, Milan, Italy) cells as a model of bronchial epithelial cells and HUVECs (CRL-1730; ATCC, Milan, Italy) as a model of endothelial cells to evaluate the presence of ACE2 and AT1R at basal levels.

HUVECs were cultured in endothelial cell growth media (Cod. #CC4176, EGMTM-2 Single Quots , Lonza, Milan, Italy) with fresh culture medium replenished every 2–3 days. When they reached confluence, 106 cells were pelleted for gene expression analysis. 

Calu-3 cells were cultured in DMEM/F-12, GlutaMAX™ supplement, (Cod. #10565018, Gibco, Milan, Italy) supplemented with 10% FBS (Cod. #ECS0180L, Euroclone, Milan, Italy) and 1% penicillin-streptomycin (Cod. #P0781, Sigma-Aldrich, Milan, Italy), with fresh culture medium replenished every 2–3 days. 

For the viral infection, in addition to Calu-3 cells, two other cell lines were used: 293T (CRL-3216; ATCC, Milan, Italy) and VeroE6 (CRL-1586; ATCC, Milan, Italy) were maintained in Dulbecco’s’ modified Eagle medium (Cod. #41965039, Gibco, ThermoFisher Scientific, Rodano, Milan, Italy). 

For the cell–cell fusion assay, Hela cells (CCL-2; ATCC, Milan, Italy) were cultured at 37 °C in a 5% CO_2_ humidified atmosphere in the Dulbecco’s Modified Eagle Medium/F-12 Nutrient Mixture Ham 1:1 (DMEM/F12) (Cod. #31331.028, Gibco, Milan, Italy), supplemented with 10% Fetal Bovine Serum (FBS) (Cod. #10270106, Gibco Milan, Italy), and 1% Penicillin-Streptomycin Solution 100X (Pen/Strep) (Cod. #ECB3001, EuroClone, Milan, Italy).

### 4.2. Cell Treatments

Before treatments, Calu-3 cells were seeded in 12-well plates at 3*10^5^ cells per well, grown to sub-confluence (80%) and starved for 16 h in serum-free medium. 

Cells were first exposed for 12 h to 100 nM Ang II (Cod. #A9525, Sigma-Aldrich, Milan, Italy), or to 10 µM of the ARB irbesartan (Cod. #I2286, Sigma-Aldrich, Milan, Italy), or to 10 µM of the ACEI ramipril (Cod. #R0404, Sigma-Aldrich, Milan, Italy), or to 10 µM of the ACE2 inhibitor MLN-4760 (Cod. #5306160001, Merck-Millipore, Vienna, AT, USA) added to fresh media 1 h before exposure to 100 nM Ang II. 

Since ACE2 converts Ang II into Ang 1-7, which acts by opposing Ang II actions in several experimental settings, we investigated the hypothesis that the effect of Ang II on ACE2 transcripts could be due to and/or modulated by Ang 1-7 formation. Hence, we exposed Calu-3 cells to Ang 1-7 (Cod. # A9202, Sigma-Aldrich, Milan, Italy) concentrations increasing from 10 nM to 10 µM. 

For SARS-CoV-2 infection assays, cells were also treated with 100 nM Dihydrotestosterone (DHT) (Cod. # 31573, Sigma-Aldrich, Milan, Italy) as a positive control of infection. 

As a proof of concept, in the cell–cell fusion assay, Calu-3 cells were also stimulated with an inhibitor of the serine-threonine protease TMPRSS2, 10 nM Nafamostat Mesylate (#N0289 Sigma Aldrich, Milan, Italy), 2 µg/mL a polyclonal antibody targeting ACE2 (cod. #ab15348, Abcam, Milan, Italy), or with 12 µg/mL soluble ACE2 recombinant protein (kindly provided by Professor Federico Forneris, Univeristy of Pavia), at least 2 h before coculturing with Hela cells.

All experiments were conducted in serum-free media because we previously detected small amounts of ACE2 in FBS. 

### 4.3. Gene Expression Analysis

Total RNA was extracted from Calu-3 cells or HUVECs with the High pure RNA isolation Kit (Cod. #11828665001, Roche, Monza, Italy) following manufacturer’s protocol. One µg total RNA was then reverse-transcribed with Iscript (Cod. # 1708841, Bio-Rad Laboratories, Segrate, Italy) in a final volume of 20 µL. Primers (*hACE2* 5′-AAAGTGGTGGGAGATGAAGC-3′, 3′-GAGATGCGGGGTCACAGTAT-5′; *hAGTR1* 5′-ATGATTCCAGCGCCTGAC-3′, 3′-GGTCCAGACGTCCTGTCACT-5′) and probes for ddPCR were designed using ProbeFinder Software (Universal ProbeLibrary, Roche). We obtained the absolute quantification of angiotensin-converting enzyme 2 (ACE2) mRNA using a Digital Droplet PCR system (ddPCR) (Bio-Rad Laboratories, Segrate, Italy). cDNA measuring 2 μL and 18 μL of the total reaction volume, containing primers, probes (Universal ProbeLibrary, Roche), and ddPCR Supermix for Probes (no dUTP) (Cod. #1863024, Bio-Rad Laboratories, Segrate, Italy), were converted into droplets with the QX200™ Automated Droplet Generator (Bio-Rad Laboratories, Segrate, Italy). Droplet-partitioned samples were transferred to a 96-well plate, and PCR was performed on the Bio-Rad C1000™ (Bio-Rad Laboratories, Segrate, Italy) with the following protocol: 95 °C for 10 min, followed by 40 cycles of 94 °C for 30 s and 60 °C for 1 min, with a final step of 98 °C for 10 min. Droplet Digital PCR data were analyzed with QuantaSoft analysis software (Bio-Rad Laboratories, Segrate, Italy). Absolute levels of the target gene mRNA were expressed as number of copies per 100 ng of retrotranscribed RNA.

### 4.4. Immunoblotting 

Immunoblotting for ACE2 was performed following a standard protocol. Briefly, Calu-3 cells were sonicated in RIPA lysis and extraction buffer, and the protein concentration was determined with BCA (Cod. #23225, Thermo Scientific, Milan, Italy). Lysate fraction (20 µg) was separated in an 8% polyacrylamide gel and electro-blotted onto nitrocellulose membrane (Cod. #10600008, Amersham-Hybond EC, GE Healthcare Life Sciences, Milan, Italy). The membranes were blocked for 1 h at room temperature in 5% Bovine Serum Albumin (BSA) (Cod. #A9430, Sigma-Aldrich, Milan, Italy) and then incubated overnight at 4 °C with an anti-ACE2 antibody (diluted 1/1000) (Cod. #ab272690, Abcam, Milan, Italy). After washing, membranes were incubated for 1 h with an antirabbit secondary antibody (Cod. #P0448; Agilent-DAKO, Milan, Italy), after which the band intensity was measured in ATOM UVITEC (Uvitec, Milan, Italy). Images were analyzed with Nine Alliance Program (Uvitec, Milan, Italy). To adjust for differences in the amount of loaded protein ACE2 expression was normalized to β-actin (Cod. #A5441, Sigma Aldrich, Milan, Italy).

### 4.5. Pseudotyping of Vesicular Stomatitis Virus 

Expression plasmids for vesicular stomatitis virus (VSVΔG-fLuc), glycoprotein (VSV-G), and SARS-CoV-2 spike protein (pCAGGS_SARS-CoV-2_spike, provided by the National Institute for Biological Standards and Control, NIBSC), used for the generation of SARS-CoV-2 pseudovirus, were previously reported [1,39,40]. VSV-ΔG-fLuc and the VSV-G expressing plasmid were kindly provided by Prof. Cristiano Salata (Dept. Molecular Medicine, University of Padua).

Vesicular Stomatitis Virus (VSV) pseudotypes were generated following published protocols [39,41]. In brief, 293T, transfected with Lipofectamine 3000 (Cod. # L3000015, ThermoFisher Scientific, Milan, Italy) to express the viral surface glycoprotein under study, was inoculated with a replication-deficient VSV vector that contains the expression cassette of firefly luciferase in place of the VSV-G open reading frame, VSV-ΔG-fLuc (kindly provided by Prof. Cristiano Salata, Dept. Molecular Medicine, University of Padua). After an incubation period of 1 h at 37 °C, the inoculum was removed, cells were washed with PBS, and fresh culture medium was added to the cell monolayer. Pseudotyped particles were harvested 16 h post inoculation and clarified from cellular debris by centrifugation and used for experiments.

### 4.6. Infection with SARS-CoV-2 Pseudovirus

Target Calu-3 cells were seeded in culture medium the day before infection (7 × 10^4^) in 24 wells plates. Complete culture medium (DMEM-F12 supplemented with 10% FBS) was removed, and cells were inoculated with SARS-CoV-2 pseudovirus (in DMEM-F12 no FBS) at a multiplicity of infection (MOI) of 0.05 for 1 h at 37 °C in a humidified incubator. One hour post infection, the medium containing the SARS-CoV-2 pseudovirus was removed, cells were washed in 1x PBS, and fresh compete medium was added to each well. Infection efficiency was quantified 16 hpi by measuring the activity of firefly luciferase in cell lysates using a commercial substrate (Cod. #6066766; Britelite plus, PerkinElmer Italia, Milan, Italy) in a plate luminometer (VictorX2, PerkinElmer, Milan, Italy). Luciferase values were normalized by the cellular protein content (Cod. #23227; Pierce™ BCA Protein Assay Kit, ThermoFisher Scientific, Rodano, Milan, Italy).

### 4.7. Generation of SARS-CoV-2 Viral Stock

For infection experiments with SARS-CoV-2, the SARS-CoV-2 isolate Milan IT (NCBI sequence MW000351.1) was propagated in VeroE6 cells. Cells were seeded (3.5 × 10^6^) in complete medium (DMEM supplemented with 10% FBS) in T175 vented-cap flasks the day before infection. Complete medium was removed, and cells were infected with the SARS-CoV-2 virus (MOI 0.01, in DMEM no FBS) for 1 h at 37 °C in a humidified incubator. The medium containing the virus was removed and replaced with fresh medium (DMEM supplemented with 2% FBS). Supernatants were collected, centrifuged at 2300 rpm for 10 min, and then stored in aliquots at −80 °C. Viral particles were titred by plaque reduction assay (PRA) as previously reported [42]. 

### 4.8. Infection with SARS-CoV-2 Virus

Calu-3 cells (27.5 × 10^3^) were seeded in 96 well plates. After 24 h, the complete culture medium (DMEM-F12 supplemented with 10% FBS) was removed, and cells were infected with SARS-CoV-2 (in DMEM-F12 no FBS) for 1 h at 37 °C at MOI 0.05. One hour post infection, the medium containing the SARS-CoV-2 virus was removed, cells were washed in 1x PBS, and fresh compete medium was added to each well. Mock controls were included in each experiment. Calu-3 cells were solvent (DMSO)-treated or treated with tested compounds for 48 h prior to infection with SARS-CoV-2. After infection, cells were washed with PBS and fresh medium containing the solvent, or the tested compounds were added. At 48 hpi, the medium was collected, and the viral titre (expressed as PFU/mL) was calculated by PRA in VeroE6 cells.

### 4.9. DSP1 and DSP2 Expression Plasmids

DSP1 and DSP2 sequences were PCR amplified from DSP1_GFP1-7_Ren1-155 and DSP2_GFP8-11_Ren156-311 in phRL-CMV derived plasmid kindly provided by Dr. Zene Matsuda [43] by using the following primers: DSP1 forward (TACGCAGCTAGCCACCATGGCTTCCAAG), DSP1 reverse (CTAGAAGAATTCTCACTTGTCGGCGGTC), DSP2 forward (AACAGGGCTAGCATGCAGAAGAAC), and DSP2 reverse (GCGAAAGAATTCTTACTGCTCGTTCTTCAG). The PCR fragments were inserted into the pcDNA3.1(+) vector using NheI and EcoRI restriction sites. The expression plasmid for the SARS-CoV-2 spike protein with the C9 tag at the C-terminus was purchased from Addgene (pcDNA3.1(+)-SARS2-spike-#145032).

### 4.10. Generation of DSP1 and DSP2 Expressing Cells

Calu-3 were seeded into a 6-well plate at 80% confluence and, after 24 h, transfected by adding 2.5 μg of the DSP1 probe and 10 μL of Lipofectamine 2000 Reagent (Cod.. #11668019, Invitrogen, ThermoFisher Scientific, Rodano, Milan, Italy) in 300 μL of Opti-MEM Reduced Serum Medium (Cod. #31985047, Gibco, Milan, Italy) per well, according to the manufacturer’s instructions. Cells were incubated 8 h at 37 °C in a 5% CO_2_ humidified atmosphere and then the medium was replaced with fresh complete growth medium.

Hela cells were seeded into a 6-well plate at 60% confluence and, after 24 h, were transfected with the standard calcium phosphate method [44]. Two mixes were prepared per well: one containing 10 μg of total DNA (for the co-transfection, the DSP2 reporter was used in 1:1 ratio with the SARS-CoV-2 spike protein or the pcDNA3.1(+) empty vector for the negative control condition) and 15 μL of 2.5 M CaCl_2_ (Cod. #C-5080, Sigma-Aldrich, Milan, Italy) in 150 μL of Milli-Q water and the other one consisting in 150 μL of HEPES buffered saline 2X (Cod. #51558, Sigma-Aldrich, Milan, Italy). The two solutions were mixed, and the obtained transfection mix was incubated for 30 min at room temperature; then, they were added to the cells. After 9 h of incubation, cells were washed three times with Phosphate Buffered Saline (PBS) (Cod. #ECB4004L, EuroClone, Milan, Italy), and fresh complete growth medium was added.

### 4.11. Cell–Cell Fusion Assay 

In order to investigate the modulation of SARS-CoV-2 entry mediated by the interaction of viral spike protein and ACE2, we used a cell–cell fusion assay, taking advantage of the system based on Dual Split Proteins (DSP) 1 and 2 developed by Matsuda et al. [43,45], where DSP1 reporter consists in the first seven β-strands of the green fluorescent protein (GFP) fused together with the first 155 amino acid residues of the Renilla luciferase enzyme (RL), while the DSP2 reporter consists of the remaining part of both GFP and RL (Figure 4 panel a and b). It is only within syncytia that DSP1 and DSP2 can self-associate, restoring a fully fluorescent GFP and a fully functional RL. 

Calu-3 cells were transfected to express DSP1 as described above. After 16 h (day 2), Calu-3 cells were seeded into a white 96-well plate with flat and clear bottom (Cod. #3610, Corning, Milan, Italy) at a density of 15 × 10^3^–20 × 10^3^ cells per well in complete growth medium. After 8 h, once cells were completely attached to the well bottom, the medium was replaced with DMEM/F12 supplemented with 0.1% FBS and 1% Pen/Strep, and cells were stimulated for 48 h with 100 nM Ang II, 10 μM irbesartan either alone or on top of Ang II. 

After a 48 h stimulation of Calu-3 cells, Hela cells expressing DSP2 and SARS-CoV-2 S protein (obtained as described above) were detached from the 6-well plate and seeded over Calu-3 cells into the 96 well plate at a density of 5 × 10^3^–20 × 10^3^ cells per well (the ratio between Calu-3 and Hela cells was 1:1). The obtained co-culture was incubated overnight to allow cell–cell fusion processes in the presence of stimuli. Cells were then washed once with PBS and incubated with 50 μL of modified Krebs Ringer Buffer (KRB) (125 mM NaCl, 5 mM KCl, 400 mM KH_2_PO_4_, 1 mM MgSO_4_, 20 mM Hepes, pH 7.4), supplemented with 0.1% D-glucose and 1 mM CaCl2 at 37 °C. Luminescence measurements were carried out using a PerkinElmer EnVision plate reader equipped with an injector unit. For each well, after 3 s, 50 μL of KRB supplemented with 5 μM coelenterazine (Cod. #sc-205904, Santa Cruz Biotechnology, Segrate, Italy) was added, and the luminescence signal (counts/second) was recorded for 30 s as the result of RL activity (representative traces in Figure 5, panel b). The relative cell-fusion values were calculated by normalizing the average RL activity of each condition to the average RL activity of the co-culture in mock conditions, which was set to 100%.

### 4.12. Statitistical Analysis and Softwares 

Statistical analysis was performed with GraphPad Prism Software™ (vers. 9.1, La Jolla, CA, USA). Median and 95% confidence interval were used to estimate variables and a two-tailed non-parametric test (Mann–Whitney test) for comparison. The same software was used to prepare the graphs herein reported. The three experimental designs reported in Figure 3 (panels a, b and c) were illustrated with Medical Art. 

## Data Availability

The data presented in this study are available upon request from the corresponding author.

## Supplementary Materials

The following supporting information can be downloaded at: https://www.mdpi.com/article/10.3390/ijms23095125/s1.
