# Supplementary material for: Angiotensin II Promotes SARS-CoV-2 Infection via Upregulation of ACE2 in Human Bronchial Cells
Source: Int J Mol Sci. 2022 May 4;23(9):5125. doi: 10.3390/ijms23095125 (PMC9102833; doi:10.3390/ijms23095125)
Supplement: Supplementary file 1 [file ijms-23-05125-s001.zip › ijms-1678598 supplementary.pdf]

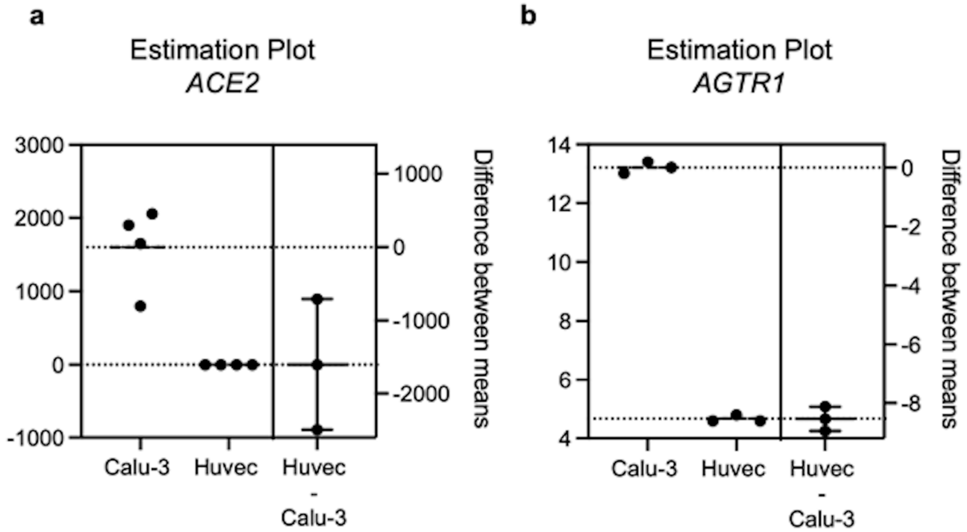

Figure S1: *ACE2* and *AGTR1* absolute mRNA levels in Calu-3 cells and HUVEC. *ACE2* and *AGTR1* absolute mRNA levels in Calu-3 cells and HUVEC were measured in basal conditions with ddPCR. Please note the much higher *ACE2* (Panel (a)) and *AGTR1* (Panel (b)) mRNA levels in Calu-3 cells than in HUVEC. Data are shown as copies/10 ng total mRNA (n=4, median ± QR, min to max).

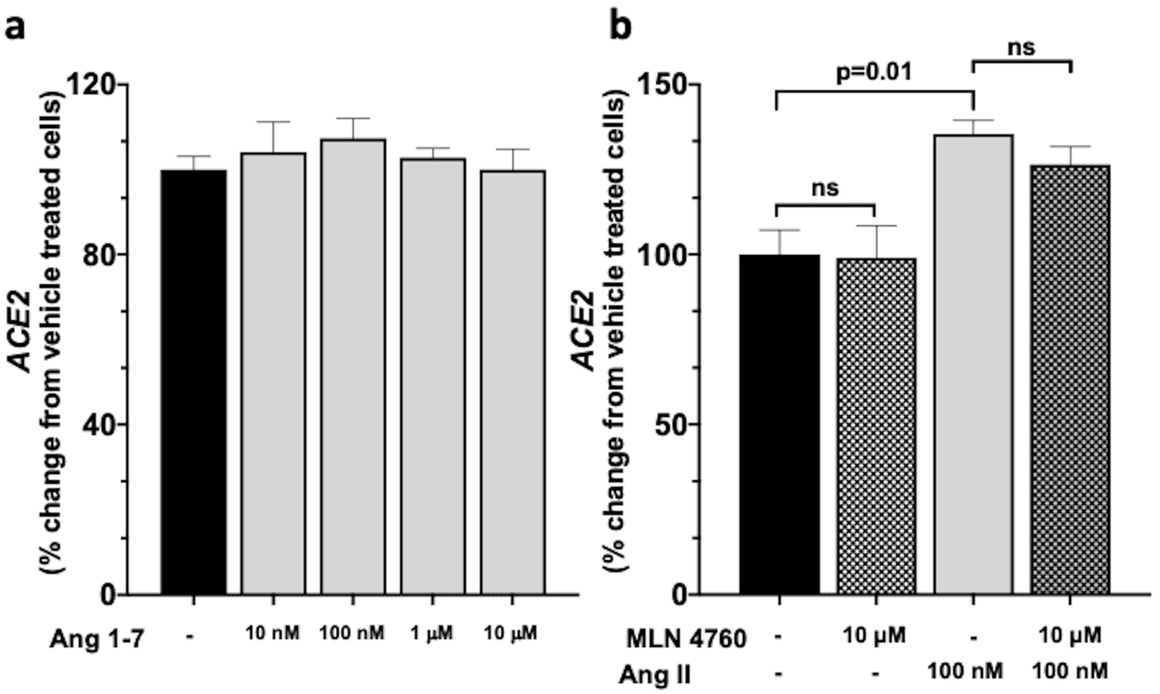

Figure S2: Ang 1-7 does not play an effect on *ACE2* gene expression in Calu-3 cells. Panel (a): The bar graph shows *ACE2* gene expression after 12-hours exposure of Calu-3 cells to increasing concentration of Ang 1-7 ranging between 10 nM and 10 µM. Ang 1-7 did not affect ACE2 gene expression. Data were measured as copies/100 ng total mRNA and then normalized *vs* vehicle treated cells (n=5 in duplicate; mean ± SEM). Panel (b): *ACE2* mRNA levels are not modulated by ACE2 blockade by MLN-4760 blockade. The graph shows *ACE2* mRNA levels after exposure of Calu-3 cells to 100 nM Ang II in presence of 10 µM MLN-4760. MLN-4760 treatment did not enhance the effect Ang II on *ACE2* gene expression (n=5 in duplicate; median ± SEM).
